# Gene co-expression network analysis reveals pathways associated with graft healing by asymmetric profiling in tomato

**DOI:** 10.1186/s12870-019-1976-7

**Published:** 2019-08-24

**Authors:** Lulu Xie, Chunjuan Dong, Qingmao Shang

**Affiliations:** Key Laboratory of Biology and Genetic Improvement of Horticultural Crops of Ministry of Agriculture, Institute of Vegetables and Flowers, Chinese Academy of Agricultural Sciences, Beijing, 100081 China

**Keywords:** Vegetable grafting, graft healing, tomato, RNA-seq transcriptome, weighted gene co-expression network analysis, co-expression profiles

## Abstract

**Background:**

The ability of severed rootstocks and shoots to re-establish vascular connections is used to generate grafted plants that combine desirable traits from both scions and rootstocks. Clarifying the mechanisms of graft healing is essential for its further application. We performed RNA sequencing of internodes near the cut position, making a distinction between separated or grafted tissues above and below the cut, in order to obtain a genetic description of graft union formation.

**Results:**

Using weighted gene co-expression analysis, variable transcripts were clustered into 10 distinct co-expression networks (modules) based on expression profiles, and genes with the most “hubness” (“hub” genes show the most connections in a network) within each module were predicted. A large proportion of modules were related to Position, and represent asymmetric expression networks from different pathways. Expression of genes involved in auxin and sugar transport and signaling, and brassinosteroid biosynthesis was increased above the cut, while stress response genes were up-regulated below the cut. Some modules were related to graft union formation, among which oxidative detoxification genes were co-expressed along with both wounding response and cell wall organization genes.

**Conclusions:**

The present work provides a comprehensive understanding of graft healing-related gene networks in tomato. Also, the candidate pathways and hub genes identified here will be valuable for future studies of grafting in tomato.

**Electronic supplementary material:**

The online version of this article (10.1186/s12870-019-1976-7) contains supplementary material, which is available to authorized users.

## Background

Many plants have the remarkable ability to regenerate tissue at a wound site and re-establish vascular connections, effectively healing the wound. When a plant is cut at the stem, the separated root and shoot parts can reunite to become a whole plant again. This healing ability is used by people to produce grafted plants for purposes of vegetative propagation or to produce novel chimeras that combine desirable traits from both the scion and the rootstock [1, 2]. Grafting is an ancient technology originally applied to tree species, although it has recently become widely used to improve yield and disease resistance in vegetable crops [3, 4]. A better understanding of the mechanisms that underlie graft healing is essential for its further application.

Physiologically, two sequential processes occur at the graft junction; callus formation to heal the wound, and tissue differentiation within the callus to reconnect the vascular strands [5, 6]. As a common theme in graft junction establishment, critical regulators are recruited for these changes. Stress-related phytohormones such as jasmonic acid and ethylene are activated within hours of wounding, triggering defense responses and callus generation [7, 8]. Cytokinin synthesis and signaling promote callus formation by manipulating cell cycle proteins [9]. In addition, the mutually exclusive interaction of auxin and cytokinin plays a pivotal role in specifying vascular patterns from the meristems [10, 11].

During graft healing, vascular components are generated asymmetrically in tissues above and below the graft junction. In Zinnia internodes, more phloem elements are formed in the upper region of the wound while more xylem elements are formed in the lower region 48 h after wounding [12]. In grafted Arabidopsis hypocotyls, reconnection of phloem elements precedes xylem reconnection by 3 days [13]. Asymmetrical behaviors of gene regulatory networks are found in the Arabidopsis inflorescence stem [14]. In the upper region of the cut gap, *ANAC071* expression is up-regulated by high levels of auxin through IAA5, ARF6, and ARF8 [15], and ANAC071 directly regulates the xyloglucan endotransglucosylase/hydrolases XTH19 and XTH20, which participate in the cell wall organization process [16]. In the lower region of the cut, *RAP2.6 L* expression is induced by low levels of auxin and jasmonic acid. In addition, ethylene slightly up-regulates *ANAC071* and down-regulates the expression of *RAP2.6 L* [14].

Concentration gradients near the cut position caused by the accumulation or depletion of some phytohormones or active metabolites, which originally show polar transport through the vascular bundles, are examples of regulatory network differences above and below the graft junction [17, 18]. Most importantly, asymmetrically distributed materials between the adhered graft partners provide inter-tissue recognition mechanisms that activate vascular regeneration [19], with auxin being the most crucial. As confirmed by mutant screening, the auxin response genes ALF4 and AXR1 act irreplaceably below the the graft junction [13]. Metabolically active sugar is also focused, as its presence is required for the induction of vascular differentiation by auxin [20, 21]. The sugar response was found to correlate with asymmetric gene expression [19].

Transcriptome sequencing has been used as an effective tool to study the genetics of graft union formation. Microarrays or RNA-seq transcriptome analyses in *Arabidopsis* and woody species such as grape, hickory, and pecan have revealed that genes involved in auxin transport and signaling, cytokinin signaling, cell proliferation and elongation, vascular differentiation, cell wall modification, wounding, secondary metabolism, jasmonic acid and ethylene biosynthesis, and reactive oxygen species (ROS) scavenging showed significant differential expression during graft union development [7, 22–24]. Research in *Arabidopsis* has described more detailed spatial and temporal dynamics [19]. In comparisons to ungrafted samples, genes that show similar high levels of expression in the four samples (grafted top, grafted bottom, separated top, and separated bottom) are mainly enriched in stress and defense responses pathways at the early stage. After 120 h, pathways related to cell wall organization and vascular differentiation were found to be upregulated in grafted samples, including both the top and bottom samples [19].

At present, grafting is widely applied to vegetable production, especially in solanaceous fruit vegetables such as tomato (*Solanum lycopersicum*), and its use is increasing. However, the molecular mechanism of graft union formation in tomato plants has not yet been investigated, expect for anatomical and physiological studies [6, 25], because the situation is likely to differ from Arabidopsis and woody plants. In this study, we collected internode stem sections from auto-grafted or separated plants, both above and below the cut position, to analyze the transcriptomic patterns during tomato graft union formation. By using Weighted Gene Co-expression Network Analysis (WGCNA) [26], specific gene co-expression networks related to graft union formation were identified.

## Results

### Phenotypic changes of the cut positions in the grafted and separated treatments

**Fig. 1:**
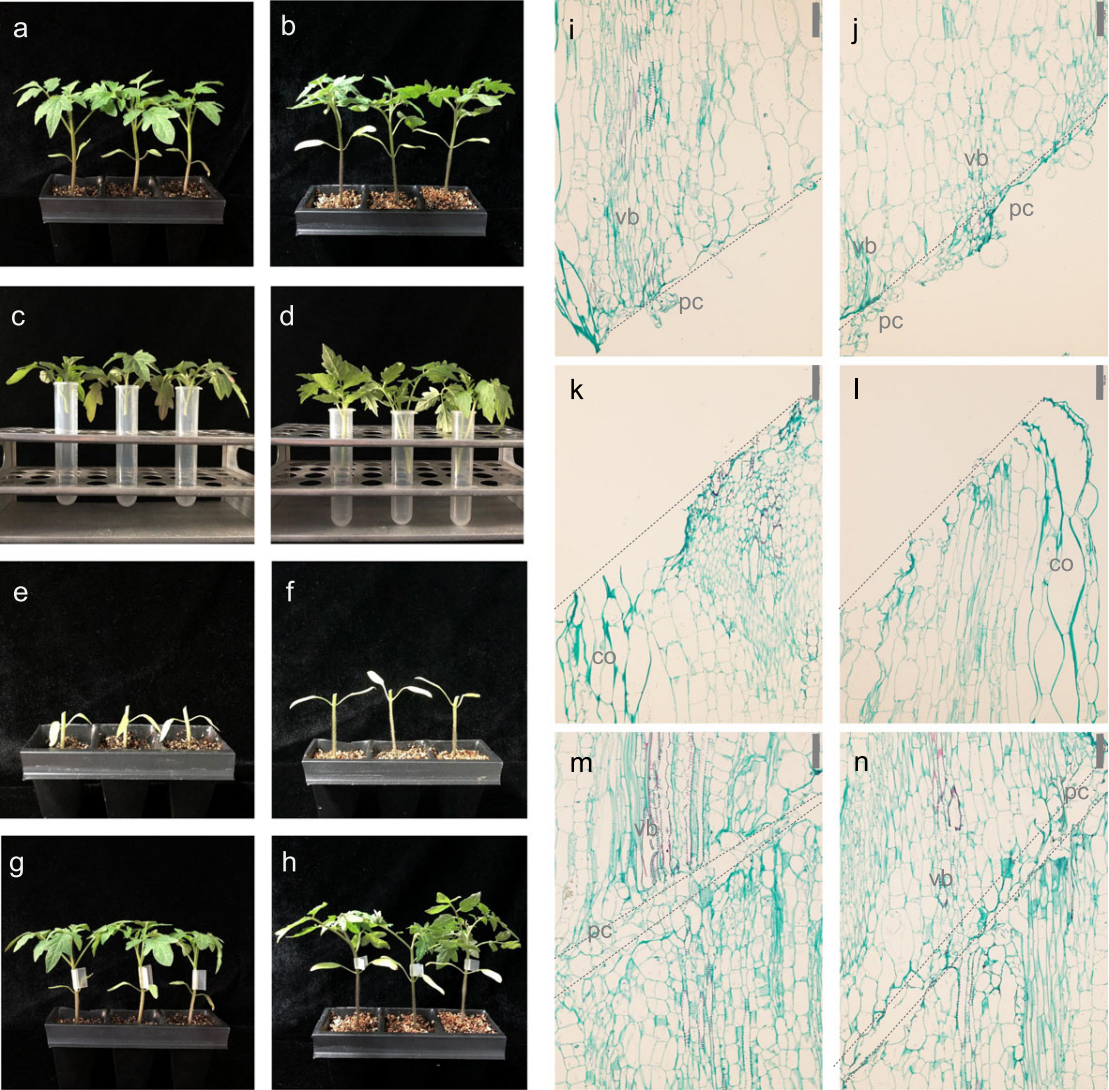
Photographs and longitudinal sections of tomato. **a**: ‘JH4’ plants before treatment; **b**: ‘JL787’ plants before treatment; **c-h**: ‘JH4’ and ‘JL787’ plants 3 days after treatment; **c**: Separated ‘JH4’ shoot parts; **d**: Separated ‘JL787’ shoot parts; **e**: Separated ‘JH4’ root parts; **f**: Separated ‘JL787’ root parts; **g**: Grafted ‘JH4’ shoot parts and root parts; **h**: Grafted ‘JL787’ shoot parts and root parts. **i-n**: Longitudinal sections near cut positions of separated shoot parts, separated root parts, and grafted plants (upper are shoot parts and lower are root parts) of JH4 (**i**, **k**, **m**) and JL787 (**j**, **l**, **n**) at 3 days after treatment . Bars at the top right corners indicate 100 μm. Dash lines indicate cut positions. Pc, vb, co are short for parenchyma cell, vascular bundle, cortex

We grew two tomato cultivars, ‘JiaHong No. 4’ (‘JH4’) and ‘JiuLv 787’ (‘JL787’) to 4-leaf stage (Fig. 1a-b). ‘JL787’ is a common rootstock cultivar in China, while ‘JH4’ is usually used as the scion. In this experiment, eight groups of plant internodes were prepared as follows (Fig. 1c-h); (1) separated ‘JH4’ shoot parts, (2) separated ‘JH4’ root parts, (3) separated ‘JL787’ shoot parts, (4) separated ‘JL787’ root parts, (5) grafted ‘JH4’ shoot parts, (6) grafted ‘JH4’ root parts, (7) grafted ‘JL787’ shoot parts, and (8) grafted ‘JL787’ root parts.

Based on our previous experience, reunion of the scion and rootstock parts of tomato seedlings could be complete < 6 days after grafting (DAG) [27]. In the autografts of both cultivars, the adhesion force between graft interfaces could be detected at 3 DAG. We therefore performed microscopic observations of internode sections near the cut position at this stage to survey phenotypic changes at the cellular level. Differences were found between the upper and lower interfaces of the separated samples (Fig. 1i-l). More parenchyma cells were observed at the upper interfaces, especially near the vascular bundles, while none were found at the lower interface. In addition, the cortex cells were unusually swollen, which could be caused by discontinuous water transport from the root to the shoot. In the grafted samples, the shoot and root parts were connected by parenchyma cells (Fig. 1m-n), which subsequently become specialized into vascular bundles.

### RNA sequencing and analysis of gene co-expression networks

**Fig. 2:**
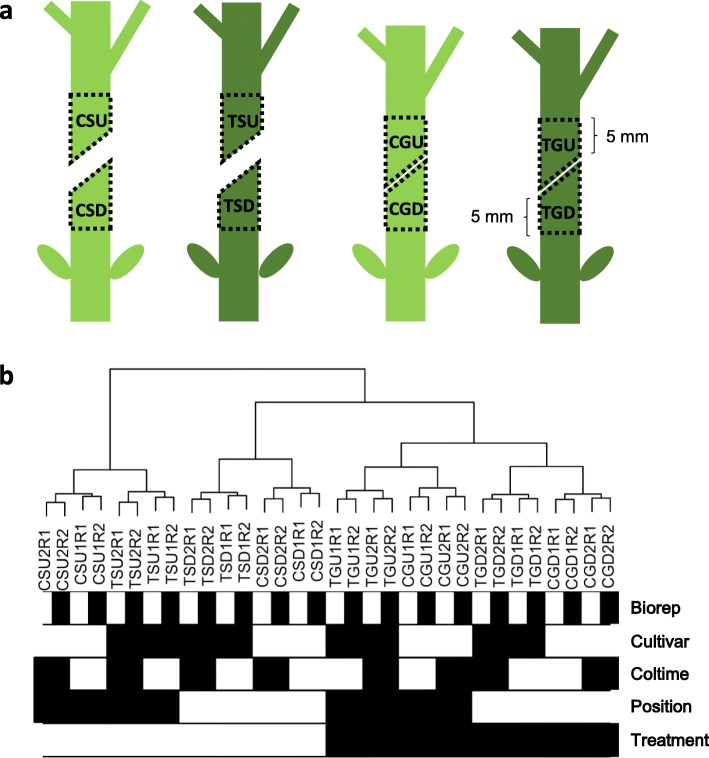
Sampling information. **a**: Schematic diagram showing the internode sections that were sampled for RNA-seq. **b**: Cluster dendrogram of different samples. The white (for 0) and black (for 1) blocks below the tree represent the two levels of the respective experimental traits

We performed high-throughput RNA sequencing to compare the transcriptome profiles that underlie the phenotypic changes. Because initial adhesion happened within 3 days after grafting, two time points of 48 h and 72 h were chosen with regard to the previous eight groups of internode sections. Each collecting with two biological repetitions. Therefore, a total of 32 RNA-seq libraries were constructed and named using the convention of Cultivar (C for ‘JH4’ and T for ‘JL787’) | Treatment (S for separated and G for grafted) | Position (U for above and D for below the cut site) | Coltime (collection time; 1 for 48 h and 2 for 72 h) | Biorep (biological replicate; R1 for replicate #1 and R2 for the replicate #2). The internode sections collected are illustrated in Fig. 2a. After sequencing and quality filtering of the raw reads, normalized FPKM values were obtained using the Tophat and Cufflinks analysis pipeline (see Methods). Mapping information is shown in Additional file 1: Table S1. Genes with low abundance and low variability were filtered out in order to reduce noise. Log2-transformed expression values of the remaining 3409 genes (Additional file 1: Table S2) were imported into the WGCNA package for co-expression network analysis. The overall relationships among the different samples is shown by a cluster dendrogram (Fig. 2b). The two levels of Biorep show good correlation. In addition, samples were clustered regularly based on all experimental traits, especially for Position and Treatment.

**Fig. 3:**
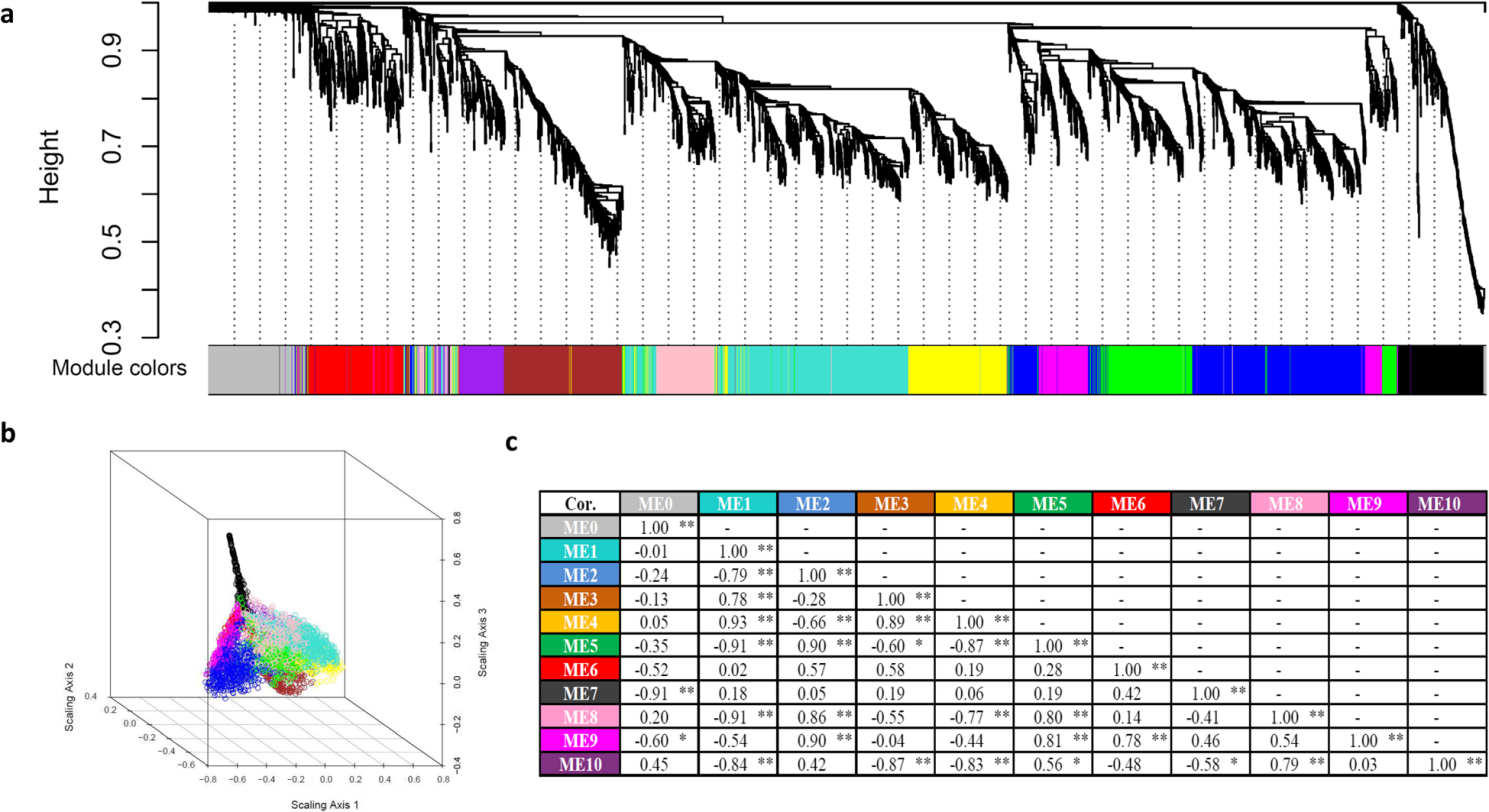
Hierarchical clustering and co-expression modules. **a**: Hierarchical cluster tree showing gene co-expression modules identified by WGCNA (Weighted Gene Co-expression Network Analysis). The branches constitute 11 modules labeled in different colors. Except for the gray module, the colored modules were named ME1 to ME10 from the largest to the smallest. **b**: 3-D multiple-dimensional scaling (MDS) plot. Each circle represents one gene, and the color of each relates to the module to which it belongs. **c**: Pairwise correlation coefficients between modules. *P*-values are indicated by asterisks; ** (< 0.01), * (< 0.05)

As a result of weighted co-expression network analysis, the expression profiles of the 3409 genes were grouped into 11 modules (MEs), reflecting 10 different co-expression networks (ME1-ME10) and the outliers that do not belong to any cluster (ME0). ME1–10 and ME0 are distinguished by different colors (Fig. 3a). The network structure was visualized using multi-dimensional scaling (MDS) scatter plots (Fig. 3b). Genes in the different modules were distributed in three dimensional space like fingers. Modules positioned close to one another are expected to have similar expression patterns.

In order to measure the relationships between different modules further, the eigengenes (the first principal component) of the modules were calculated in each sample set (Fig. 3c). Some module pairs indicated by eigengenes are significantly positively or negatively correlated. For example, ME1 (turquoise) is positively correlated with ME3 (brown) and ME4 (yellow), while ME1 is negatively correlated with ME2 (blue), ME5 (green), ME8 (pink), and ME10 (purple). ME9 (magenta) showed a significant positive correlation with ME2, ME5, and ME6 (red). In addition, ME7 (black) showed no correlation with any of the other colored modules.

### Certain co-expression networks show obvious correlations with certain experimental traits

**Fig. 4:**
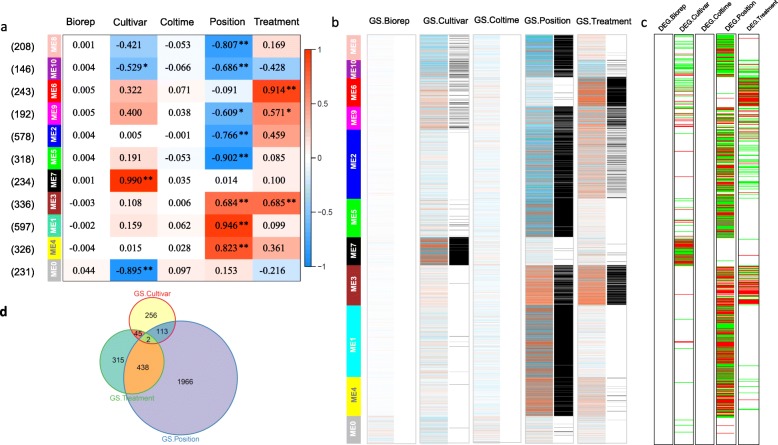
Relationships between modules or individual genes and Traits. **a**: Relationships between modules (left) and Traits (top). The numbers in brackets on the left show the number of genes in a module. Red and blue represent positive and negative correlations, respectively, with coefficient values on. The darker colors, indicate higher correlation coefficients. *P*-values are indicated by asterisks; < 0.01 (**), < 0.05 (*). **b**: Relationships between individual genes and Traits. For each trait, the left columns colored in red (positive) and blue (negative) are Gene Significances (GSs), and the right columns colored in black (significant) and white (insignificant) are Significance judgements. **c**: Differentially expressed genes (DEGs) of each trait. Columns colored in dark red (positive) and green (negative) are Log2FC values of significantly differentially expressed genes. **d**: Venn diagram of genes that are significant with respect to Cultivar, Position, and Treatment. The areas in each circle are proportional to the numbers in each gene set

When associating the co-expression networks with certain experimental traits, diverse correlation patterns indicated by module eigengenes were obvious (Fig. 4a). As expected, the biological replicates did not display correlations with any module. Besides, there were no significant correlations in the Coltime column, indicating that there were no considerable changes in the gene expression profiles at 48 h and 72 h after treatment. Obviously, most of the modules showed strong correlations with Position. There were positive correlations between Position and ME1, ME3, and ME4, implying the presence of up-regulated eigengenes in these modules in the section above the cut site. In contrast, there were negative correlations between Position and ME2, ME5, ME8, and ME10, and ME9 had a lower coefficient value but the correlation was still significant. We found that no modules were negatively correlated with the most important factor, Treatment, while ME3 and ME6 showed marked positive correlations, suggesting that graft treatment may induce increased expression levels in groups of genes. Therefore, by eigengenes, we found integral features of gene co-expression networks that were related to every experimental trait.

At the same time, we also focused on the expression profiles of individual genes. We made use of Gene Significance (GS) measures, the coefficients of the Pearson correlations between individual genes and trait factors. The higher the absolute GS value, the more biologically significant is the gene. GS provides detailed information, which is especially important in the unsigned network. As shown in Fig. 4b, the GSs exhibited diverse patterns when placed in the context of the modules. The color intensities in the matrix are generally consistent with the module eigengene-trait image. Also, no GS was detected for Biorep and Coltime. The GSs for Cultivar were found mainly in ME7, with very few in ME0. As for Position, ME1, ME3, and ME4 had more positive GSs, while ME2, ME5, ME8, ME9, and ME10 had more negative GSs. In addition, the GSs for Treatment clustered mainly in ME3 and ME6, with fewer in ME9, implying biological significance for the graft treatment for the genes in these modules.

As WGCNA is a method to get a broad view of the gene expression levels in the network scale, we also performed pair-wise differential expression tests in parallel to co-expression network analysis. Differentially expressed genes were shown in Fig. 4c, corresponding to the arrangement order of individual genes in Fig. 4b. It was obvious that the GSs from co-expression network analysis and the DEGs from pair-wise tests had a very similar profile. Therefore, GSs were reliable to reflect expression patterns of particular genes.

Venn diagrams were used to visualize the overlaps between GSs of the Cultivar, Position, and Treatment traits. As shown in Fig. 4d, GS for Position comprised the largest circle, implying that the largest number of genes were differentially expressed between internode sections above and below the cut site. With the intersection areas weighted by gene numbers, it was clear that the GSs for Position and Treatment overlapped considerably with each other.

### Significant biological functions are enriched in the different co-expression networks

**Fig. 5:**
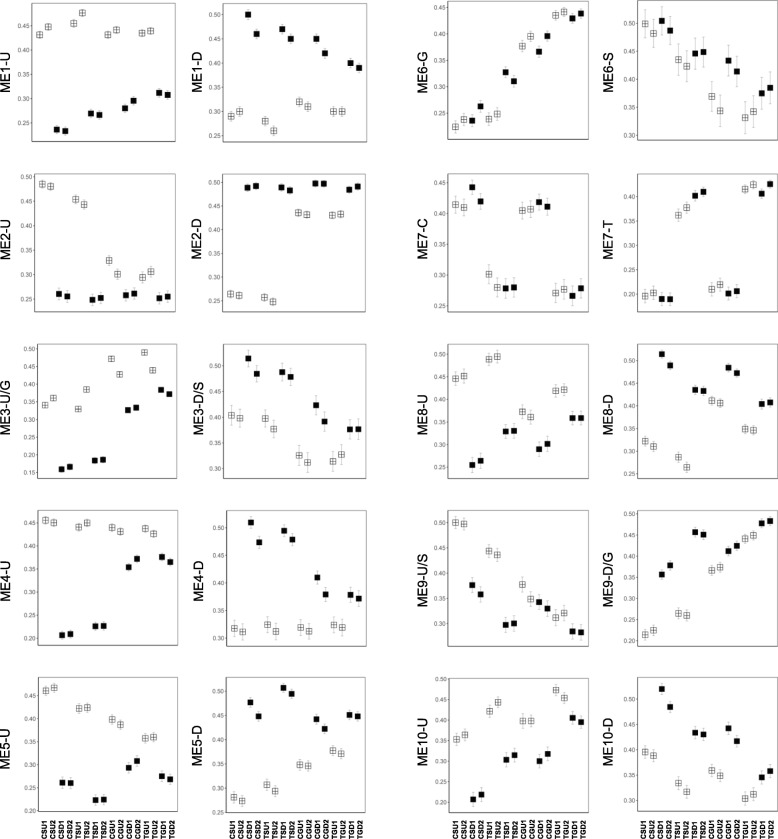
Mean 0–1 transformed log2 FPKM expression profiles. Expression profiles are shown as two parts in each module, divided based on the different mean levels of the given traits. Samples with U and D properties are represented by open squares and solid squares, respectively, with standard error bars

Based on the previous results, the expression profiles of genes in modules and focused traits were displayed. Because all trait factors had two levels, genes in each module were separated into two parts, often with opposite modality (Fig. 5). For example, because ME1 was significantly related to the factor Position, genes with average values in the U samples that were larger than the average values in the D samples were labeled as ME1-U, and the remaining were labeled as ME1-D. We considered that both parts have biological meaning. Accordingly, GO annotation and enrichment analyses were performed (Fig. 5, Additional file 1: Table S3, Additional file 2: Figure. S1). Tomato genes were first used as queries. When the tomato database was not adequate, Arabidopsis ortholog genes were then used as queries. GO terms derived from the two annotations are given in Additional file 1: Table S3.

As can be seen from ME1-U and -D, the expression levels of the U and D samples diverged dramatically when they were separated, and the divergence was abbreviated from both sides when grafted. The most enriched functions in ME1-U included sterol biosynthesis, water transport, and mitotic cell cycle. And in ME1-D, except for cell redox homeostasis and xyloglucan metobolism, more items were found in annotations of Arabidopsis orthologs, including response to chitin, photomorphogenesis, response to ethylene, response to jasmonic acid, response to wounding, and organic anion transport.

In ME2-U, the expression levels of all the D samples were consistently low, and the U sample levels in the separated treatments were high, but were low in the grafted treatments. Biological processes like water deprivation, inorganic anion transport, plant organ senescence, lipid storage, and starch metabolic process were included. In ME2-D, the expression levels of all the D samples were consistently high. Genes that participate in energy and defense functions, such as photosynthesis, response to other organism, ROS metabolism, and toxin metabolism were enriched, and they showed consistently high expression in the D samples and became closer to the U samples when grafted.

In addition, expression levels in ME4-U remained high in the U samples. Few transcripts were in the separated D samples, but the number became closer to the U samples when grafted. Biological processes related to auxin transport and signaling were found to be enriched in this group of genes. In the separated samples in ME4-D, hexose biosynthesis and serine family amino acid metabolism were enriched.

Gene expression profiles in ME5, ME8, and ME10 were similar to ME1, with minor differences. Of the first two modules, sterol metabolism was the main term in the U samples, and photosynthesis was the main term in the D samples. In the ME10 module, acetyl-CoA metabolism and positive regulation of cell communication were enriched in the U and D samples, respectively.

ME3, ME6, and ME9 were the modules related to Treatment. Although ME3 and ME9 were related to the Position trait, grouping by Position and by Treatment resulted in the same two sets of genes. Uniformly, ME3-D/S, ME6-S and ME9-U/S, with S (separated) characteristics, were found to be enriched in either no or very few functions. In contrast, G samples (grafted) were found to be enriched in some specific functions in the different modules. As in ME3-U/G, genes with functions associated with cell wall biosynthesis were significant in the U samples when grafted. In ME6-G, except for L-phenylalanine or lignin metabolic processes, the most significant biological processes were related to detoxification, such as response to toxic substance, hydrogen peroxide catabolism, and response to oxidative stress. The situation was similar in ME9-D/G. These results suggest that the biological processes cell wall biosynthesis and detoxification are important in internode sections of the grafted samples.

ME7 was the only module associated with the variable Cultivar. Genes with higher expression levels in T samples (from ‘JL787’) were enriched in L-phenylalanine metabolic processes and the production of siRNA involved in RNA interference compared to the C samples (from ‘JH4’). This may imply that the rootstock cultivar ‘JL787’ has stronger biotic or abiotic resistance than does the scion cultivar ‘JH4’. Except for this, the gene expression patterns of the two cultivars were similar in all of the other co-expression clusters. However, the number of differentially expressed genes used to measure the cultivar related-effect was limited even for the two selected cultivars, because some germline-specific reads were ignored when mapping to the tomato reference genome in this work. Therefore, a more detailed effort should be made to investigate the cultivar-related effect.

**Fig. 6:**
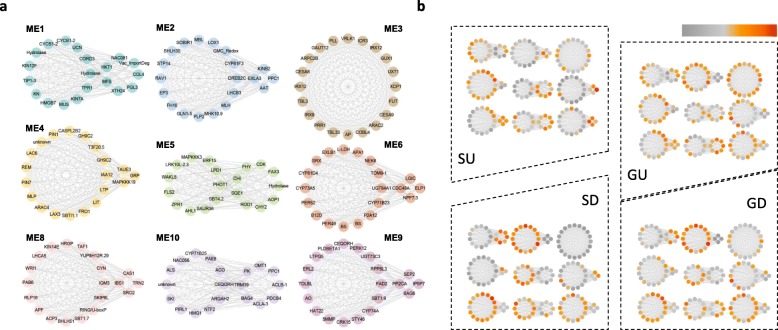
Hub genes and expression profiles. **a**: Co-expression gene networks with the greatest hubness in every module. Nodes are represented by dots coated with module colors. Two circles in one module indicate genes with reverse expression patterns. **b**: Mean expression profiles in SU, SD, GU, GD samples of hub genes. The locations of each gene were corresponding to (**a**). The darker the orange color, the higher the expression level

In order to view the detailed expression patterns of pivotal individual genes, we assigned hub genes in each module. The top 20 genes with the largest hubness were selected to represent the modules (Fig. 6). The biological functions in which the hub genes participate were generally in agreement with the enriched GO terms, but provided more detailed information. For example, ME4 was enriched in the auxin signaling and efflux pathways, and included the candidate hub genes *PIN1* (Solyc03g118740.2), *GH9C2* (Solyc12g055970.1) and *IAA9* (Solyc04g076850.2).

## Discussion

### Graft union formation-specific co-expression networks were dissected from the position effect of scion and rootstock using the WGCNA method

In the present work, we undertook an effort to learn more about the molecular events of grafting in tomato by using RNA-seq transcriptome and WGCNA analyses. By progressive steps, we first divided the variable genes into co-expression modules by unsigned network construction based on their expression patterns. The modules were then associated with given traits, and gene hubness was used to construct the relationships between individual genes and the position or grafting variables. The present analysis pipeline was able to handle the 32 sample dataset and consider the multiple traits together. First, unsigned network construction, which resulted in bi-directional changes in a module, is more reasonable than signed. Because systematic biological reactions always exhibit trade-offs, the reverse profiles of some branched pathways were caused by changes in the main effect [28, 29]. When genes with a certain change direction, reflected by average values, were put in an annotation database, whether or not there were significant terms was used as a test to distinguish the main effect from reverse trade-off, because the contributors of main effect prefer to be recruited in a functional core [30, 31]. This also can explain why many of the modules in Fig. 5 and Additional file 1: Table S3 have significantly enriched terms only in one of two parts of the genes. Second, eigengenes and hub genes in each module provide convenience to establish relationships between co-expression clusters and focused traits in order to obtain clear expression patterns and provide candidate genes.

By using this method, graft union formation-specific co-expression networks were dissected from the other traits, especially the position effect on the scion and rootstock. Among the three gene clusters with elevated expression levels in grafted internodes, only one was associated with treatment (ME6-G), from which we can obtain the biological functions that were only triggered by grafting. The other two were also related to position effect, implying that graft formation mechanisms likely originated from the scion or the rootstock. Together, this suggests that graft union formation is either influenced by the different positions of the graft union, or has developmental characteristics independent of the scion/rootstock.

### Transcriptional differences along with phenotypic changes suggest that asymmetric patterns exist between the scion and rootstock

Studies in *Arabidopsis* showed that asymmetric molecular events occur near the cut position between the scion and rootstock parts, and provide inter-tissue recognition mechanisms as shown by densely-sampled transcriptome analysis [19]. In the present study, even with limited time points, a similar situation was identified in tomato internodes, with slight differences.

Coincident with the phenotypic changes where more parenchyma cells are generated at the cut surface of separated scions, several associated pathways were revealed, including cell cycle processes and brassinosteriod biosynthesis (Fig. 5, Additional file 1: Table S3, ME1-U). More active cell divisions could be promoted by the accumulation of metabolic sugar (ME2-U) and auxin (ME4-U) [20, 21], which are derived from the shoots. Once the cells of the scion become attached to the rootstock, sugars are carried out from tissues above the cut position to tissues below the cut position [19]**.** So there are fewer parenchyma cells when grafted. Furthermore, although not reflected in the phenotype, transcripts of auxin efflux and signaling genes, and phloem and xylem pattern formation genes were enriched in tissues above the cut position. Moreover, grafting induced gene expression in the tissue below the graft junction that approached the high level of expression in the tissue above the graft junction, recruiting *PIN1* (Solyc03g118740.2), *GH9C2* (Solyc12g055970.1), and *IAA9* (Solyc04g076850.2) as candidate hub genes (Fig. 6), implying a canalization role for auxin against the tissues below the graft junction.

Brassinosteriod hormones are well known for their activities in controlling growth and the stress response [32]. It has been reported that there is crosstalk between the brassinosteriod signaling pathway and auxin via co-regulation of IAA response genes like *IAA* and *GH3*, but with slow action [33]. Moreover, brassinosteriods also interact with jasmonic acid in several biological processes such as root development and anthocyanin accumulation [34, 35]. As shown in the results, genes for brassinosteriod biosynthesis (ME1-U), auxin signaling (ME4-U), and jasmonic acid response (ME2-D) were present in different modules, suggesting that these three events are independent. Clarifying the crosstalk between brassinosteriod signaling and auxin during graft union formation maybe help elucidate the mechanisms underlying the phenotypic changes.

### Expression of oxidative detoxification processes are correlated with multiple modules during graft union formation

As shown previously by physiological studies in tomato, oxidative detoxification enzymes accumulate to high levels near the graft region [25]. Our results demonstrated that genes involved in processes such as hydrogen peroxide catabolic process (GO: 0042744), response to oxidative stress (GO: 0006979), and cellular oxidative detoxification (GO: 0098869) were enriched in two co-expression modules with independent expression profiles (Fig. 3c, Fig. 5, Additional file 1: Table S3). The first was ME2-D, in which expression was consistently high in both grafted and separated bottom tissues, and mRNA abundance was greater in grafted scions than in separated scions. Other pathways in this cluster included jasmonic acid and ethylene responses, and immune and defense responses. ROS have been reported to accumulate shortly after wounding and to participate in many stress responses [36, 37]. This explains why the first group of oxidative detoxification genes had co-expression relationships with genes involved in wounding and defense responses.

The second module was ME6-G, in which genes were expressed at higher levels in grafted tissues than in separated tissues, together with L-phenylalanine and lignin metabolism genes. Phenolic compounds are known to be biochemical markers of incompatible grafts without vascular bundle reconnection [38]. The phenolics are deposited in the cell walls of compatible combinations, but are transported into vacuoles in incompatible combinations [39]. Lignin is required for secondary thickening of the cell wall, and the lignin biosynthesis pathway is expressed normally in grafted tissues. Also, timely scavenging of ROS by peroxidase was found to be necessary to promote graft formation [25, 40], and incompatibility is accompanied by decreased peroxidase activity [41]. Thus, the co-expressed oxidative detoxification may be necessary to ensure an ordered rearrangement of phenolic compounds.

The multifaceted expression patterns of oxidative detoxification processes suggest either independent or linked functions during the wounding response and phenolic compound rearrangement. This process may act as an important switch point in graft union formation. Because we were limited to only a few sample collection times, detailed gene expression profiles cannot be obtained from the data generated in this study. Therefore, a longer experiment with more sample collection time points will be required in future studies of gene expression during graft healing.

## Conclusions

In this work, we performed gene co-expression network analysis by using RNA sequencing data from separated or grafted internode tissues above and below the cut to reveal co-expression modules during graft healing in tomato. Most of the co-expression modules were related to Position, and represent asymmetric expression profiling of different pathways. Expression of genes involved in auxin and sugar transport and signaling, and brassinosteroid biosynthesis was increased above the cut, while stress response genes were upregulated below the cut. Some modules were related to graft union formation, such as L-phenylalanine and lignin metabolic processes, among which oxidative detoxification genes were co-expressed along with both wounding response and cell wall organization genes. The present work provides a comprehensive understanding of graft healing-related gene networks in tomato and candidate hub genes for future studies.

## Methods

### Plant materials and culture

Tomato cultivars ‘JiaHong No. 4’ (‘JH4’) and ‘JiuLv 787’ (‘JL787’) were used in the experiments. Seeds of these cultivars were obtained commercially from Jingyan Yinong (Beijing) Seed Sci-Tech Co., Ltd. and Beijing Yuzhengtai Seed Co., Ltd. in China, respectively. Seedlings were grown in a greenhouse at the Institute of Vegetables and Flowers, Chinese Academy of Agricultural Sciences, Beijing. At the 4-leaf stage, the internodes between the cotyledon and the first true leaves were cut at an angle of 45 degrees. After cutting, one set of tomato seedlings had their shoot parts and root parts separated from each other and and were laid vertically as in their original status (“separated treatment”). Another set of cut seedlings were reattached and fixed with grafting tubes (“grafted treatment”). All plants were transferred to a dark culture chamber for 48 or 72 h at a constant temperature of 26 °C with 95% relative humidity.

### Microscopic observation

Internode stem sections (5 mm in length) above or below the cut position of the “separated” or “grafted” samples were excised and fixed in 50% FAA (formaldehyde:acetic acid:ethyl alcohol (50%), 5:5:90). Paraffin sections (4 μm) were prepared by vacuum infiltration, washing, gradient dehydration, clearing, paraffin embedding, and sectioning. Tissue samples were sectioned continuously in the longitudinal direction until the vascular bundle was reached. The tissue sections were stained with Safranin O and Fast Green and were observed with a light microscope (Olympus BX53).

### Sample collection and RNA sequencing

Internode stem sections (5 mm long) above and below the cut position of “separated” or “grafted” samples were collected and pooled for RNA extraction. Each pool included 20 sections. Total RNA was isolated with TRIzol reagent (Invitrogen) from each sample pool.

The mRNA-seq libraries were constructed using the Illumina TruSeq RNA Sample Preparation Kit (Illumina) with insert sizes of 200 ± 25 bp for each sample according to the manufacturer’s instructions. The libraries were then sequenced on the Illumina HiSeq 2000 platform to generate paired-end transcriptome reads (Novogene Bio Tech Co. Ltd). The raw data have been submitted under BioProject number PRJNA528328 to the Sequence Read Archive (SRA) database at NCBI.

### Transcriptome analysis

The *S. lycopersicum* reference genome sequence (v2.4) and annotation files were downloaded from the Phytozome database [42]. To process the raw sequencing reads, we used NGSQCToolkit v2.3.3 [43] to remove the paired-end reads containing ambiguous bases (Ns) or reads in which the number of low quality bases (Phred quality score < 20) exceeded 30% (−l 70, −s 20). The first unstable 10 bases were trimmed from the filtered reads. The clean reads were then mapped to the *S. lycopersicum* reference genome sequence using Tophat v2.0.9 with the default settings [44]. After duplications were removed by SAMtools v0.1.19 [45], the remaining reads were assembled referring to the general feature format files. The relative abundances (fragments per kilobase of exon per million fragments mapped; FPKM) for every transcript in each sample were estimated and normalized by Cuffquant and Cuffnorm in the Cufflinks software package (v2.2.1) [46]. FPKMs were scaled via the median of the geometric means of fragment counts across all libraries.

### Selection of variable genes

Genes in which the expression varied between different samples were selected with the FindVariableGenes function in the SEURAT package [47]. Genes were first divided into 20 bins based on their average expression levels across all samples. The log-transformed values of the variance divided by the mean, as a measure of dispersion, were then calculated in each bin and *z*-scored. A set of variable genes were then obtained by setting the threshold of the dispersion value > 0.8 and the average log2-transformed FPKM value > 3 (Additional file 3: Figure S2).

### Gene co-expression network analysis

The RNA-seq data was analyzed for gene co-expression networks using the R package WGCNA [26]. First, the PickSoftThreshhold function was used to choose a soft threshold (power) value by applying the approximate Scale-free Topology Criterion. We produced the scale-free topology fitting indices for different powers. A suitable power was chosen when the scale-free topology fit did not improve after increasing the power, and with a signed R^2^ threshold > 0.8. Here the value was 16 (Additional file 4: Figure S3). We then used the automatic network construction function blockwiseModules to obtain weighted co-expression clusters, called modules, with the following settings for the calculation processes: power = 16, networkType = unsigned, corType = pearson, minModuleSize = 100, and mergeCutHeight = 0.05.

Gene Significance (GS), expressed as the coefficients of the Pearson correlations between gene expression and trait factors, were used to associate individual genes with the traits. *P*-values of the correlations were obtained by Student’s t-test, and adjusted by the False Discovery Rate (FDR) method. FDR values < 0.01 were selected for the following analyses. A Chow-Ruskey venn diagram of significant differentially expressed genes was constructed in the R package Vennerable [48].

Module eigengenes, defined as the first principal component of the expression matrix, were used for summarizing the module profiles, and are also correlated with the trait data. The eigengene-based connectivity *k*_*ME*_ for each gene was calculated based on the Pearson correlation between the expression level and the module eigengenes by the function signedKME. Hub genes are those that show the most connections in the network as indicated by their high absolute *k*_*ME*_ value. The numbers of edges are also used for measuring hubness; the more edges of a node, the higher the hubness of the gene. The edge numbers were positively related to the absolute *k*_*ME*_ values. Hub genes were assigned according to both the *k*_*ME*_ value and edge numbers.

### Pair-wise differential gene expression tests

Comparations of gene expression profiles were performed between two levels of each trait by DESeq2 [49]. All gene counts data of 32 samples were used as input. The differentially expressed genes (DEGs) were identified with adjusted *P*-values less than 0.01. Log2-transformed fold change (Log2FC) values (Biorep-R2/R1, Cultivar-T/C, Coltime-2/1, Position-U/D, and Treatment-G/S) were calculated.

### Gene annotations and enrichment analysis

GO annotation of biological processes and overrepresentation analysis were performed in AmiGO [50] and ClueGO [51]. The genome stable ID of *S. lycopersicum* was used as a query in AmiGO annotation. Tomato sequences were used as search queries against the *Arabidopsis thaliana* reference genomes (TAIR10) using the BLASTP program [52]. The best *Arabidopsis* hits were then used as queries in ClueGO to visualize the networks.

As in AmiGO, all gene databases of *S. lycopersicum* and *A. thaliana* were used as reference lists (GO ontology database released 2018-09-06). The genome stable IDs of each gene set were used as analyzed lists. *P*-values were derived from Fisher’s exact test, and then corrected by FDR calculation. GO terms with FDR values < 0.01 were selected to output.

The Cytoscape plug-in ClueGO was used as another GO annotation tool (GO ontology database released 2018-09-06), because it is convenient for displaying the hierarchical relationships and removing redundant GO terms. The ClueGO networks were set to ‘medium’ and their connectivity was based on a kappa score of 0.4. Two-sided hypergeometric tests were applied, and *p*-value correction was performed using the Bonferroni step-down method. GO terms with adjusted *p* ≤ 0.01 were considered to be significant. The GO term grouping parameters set the initial group size at 1 and group merge at 50%, with the leading group terms based on Highest Significance.

## Additional files

Additional file 1 **Table S1.** Mapping information of the 32 transcriptoms. **Table S2**. Log2-transformed FPKM values of the 3409 variable genes. **Table S3.** Significant GO terms of each gene set. (XLSX 1705 kb)

Additional file 2 **Fig. S1.** Significant GO terms and ontological relationships (derived from the ClueGO annotation). The size of the circles are positively related to the significance of the GO terms. Redundant terms were grouped and presented with same colors, and each leading term (with highest significance) was labeled by colored font. (PDF 795 kb)

Additional file 3 **Fig. S2.** Dispersion pattern of gene expression data among the different RNA-seq samples. Dots labeled with names indicate the variable genes selected. (PDF 356 kb)

Additional file 4 **Fig. S3.** Soft thresholds chosen by applying the approximate Scale-free Topology Criterion. (PDF 38 kb)

## Data Availability

The datasets used during the current study are available from the Sequence Read Archive (SRA) database at NCBI under BioProject number PRJNA528328.

## Abbreviations

DEG: Differentially expressed gene
FDR: False Discovery Rate
FPKM: Fragments per kilobase of exon per million fragments mapped
GS: Gene Significance
Log2FC: Log2-transformed fold change
MDS: Multiple-dimensional scaling
MEs: Modules
ROS: Reactive oxygen species
WGCNA: Weighted Gene Co-expression Network Analysis
