# Supplementary figures and images for: Gene co-expression network analysis reveals pathways associated with graft healing by asymmetric profiling in tomato
Source: BMC Plant Biol. 2019 Aug 24;19:373. doi: 10.1186/s12870-019-1976-7 (PMC6708225; doi:10.1186/s12870-019-1976-7)

ME1-U

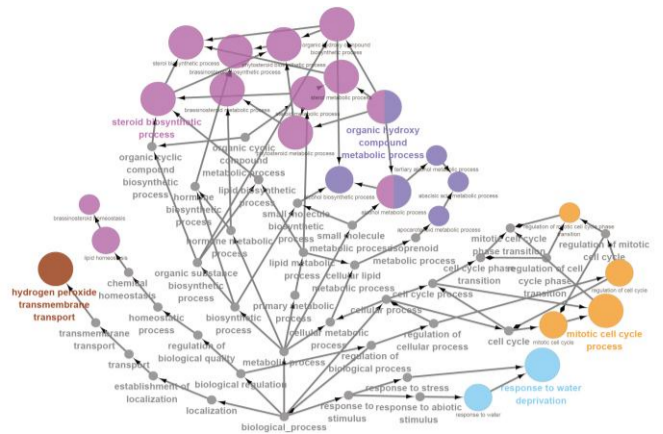

ME2-U

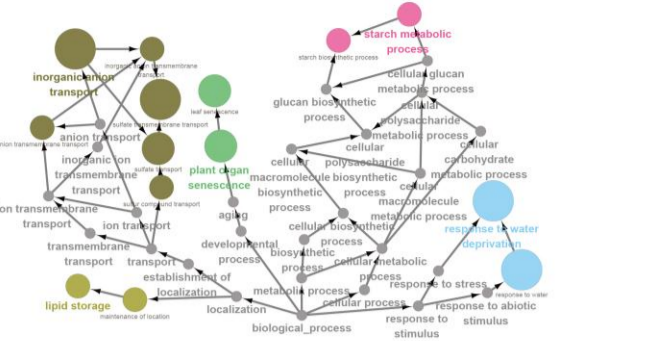

ME4-U

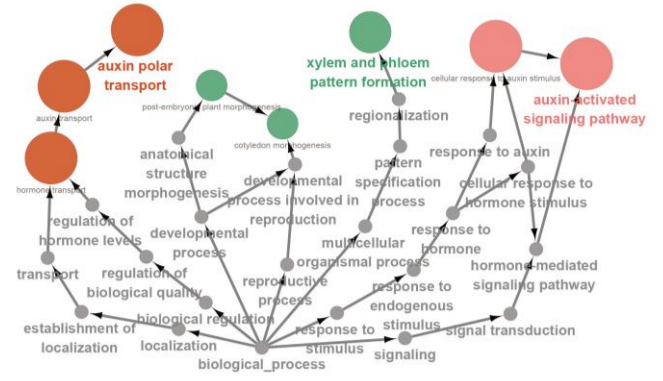

ME1-D

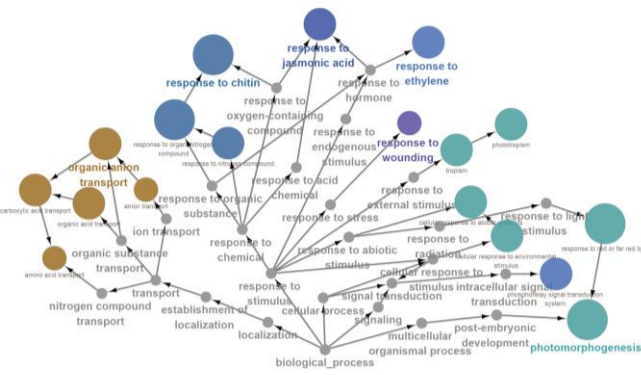

ME2-D

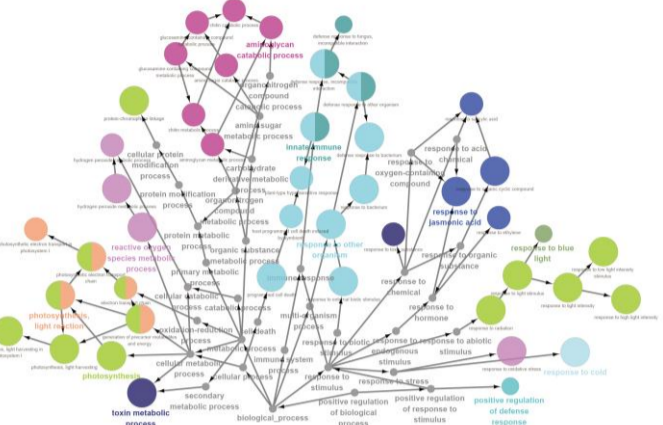

ME4-D

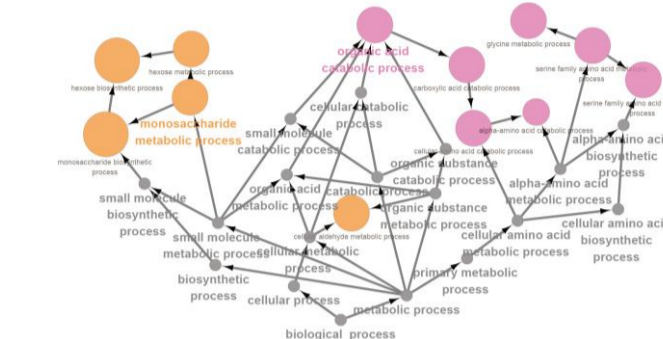

ME5-U

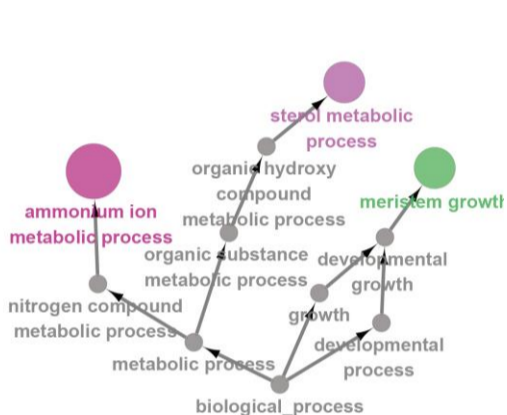

ME8-U

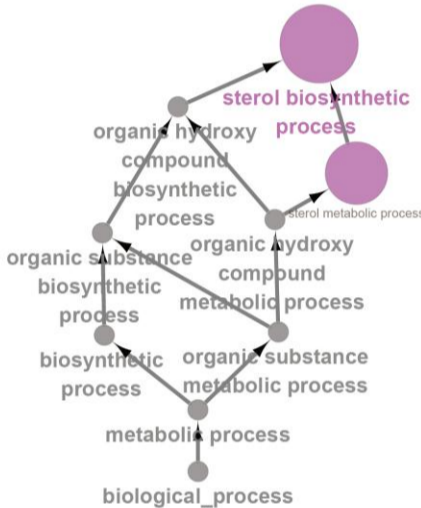

ME10-U

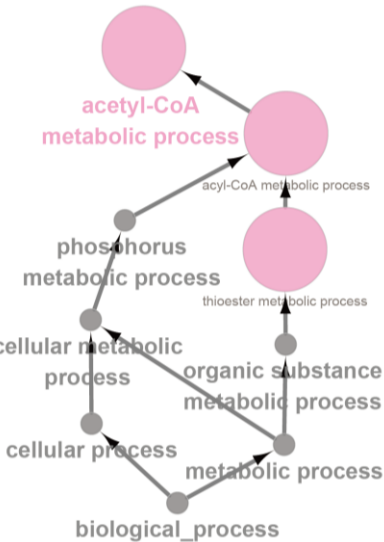

ME5-D

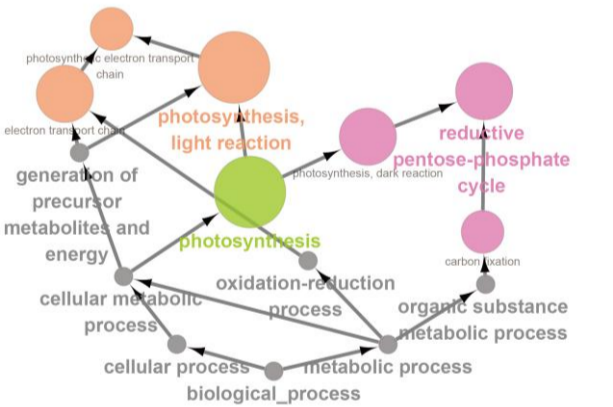

ME8-D

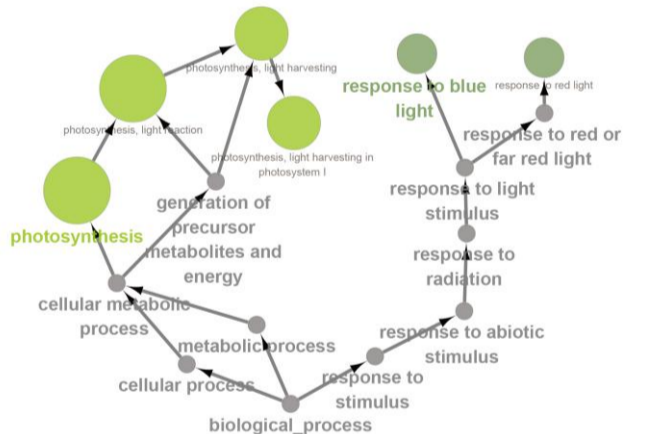

ME10-D

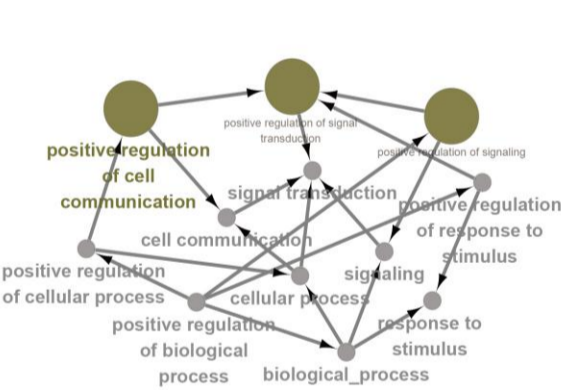

ME3-U/G

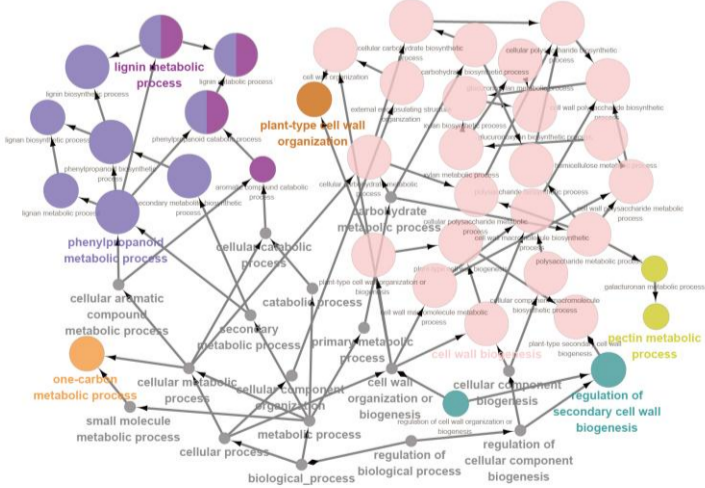

ME9-D/G

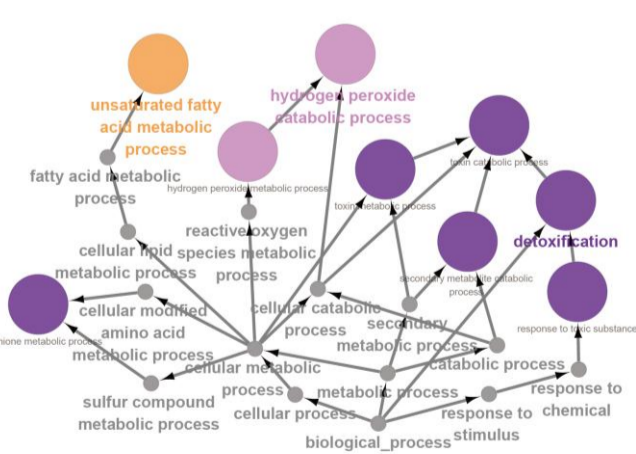

ME7-T

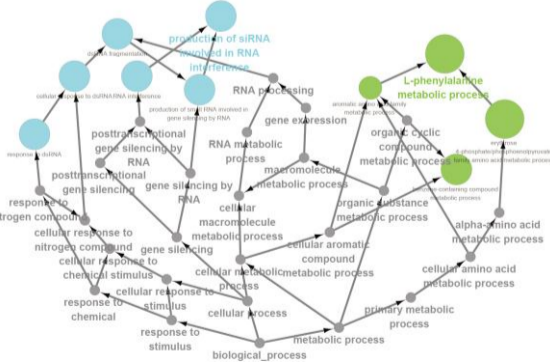

ME6-G

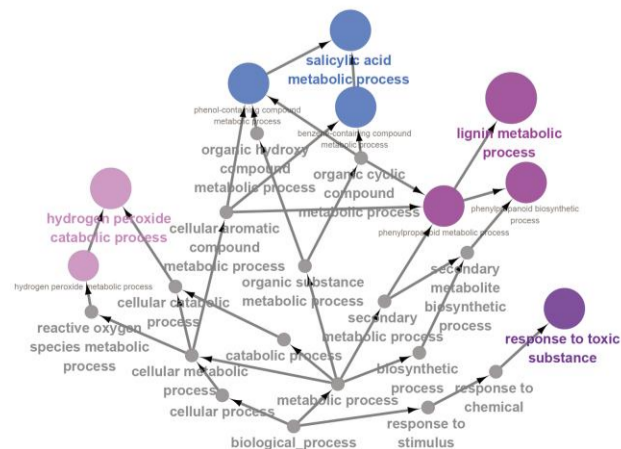

ME9-U/S

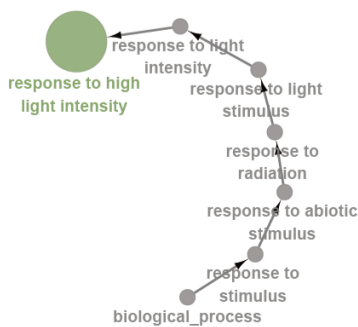

Supplement: Supplementary file 2 — Fig. S1. Significant GO terms and ontological relationships (derived from the ClueGO annotation). The size of the circles are positively related to the significance of the GO terms. Redundant terms were grouped and presented with same colors, and each leading term (with highest significance) was labeled by colored font. (PDF 795 kb) [file 12870_2019_1976_MOESM2_ESM.pdf]

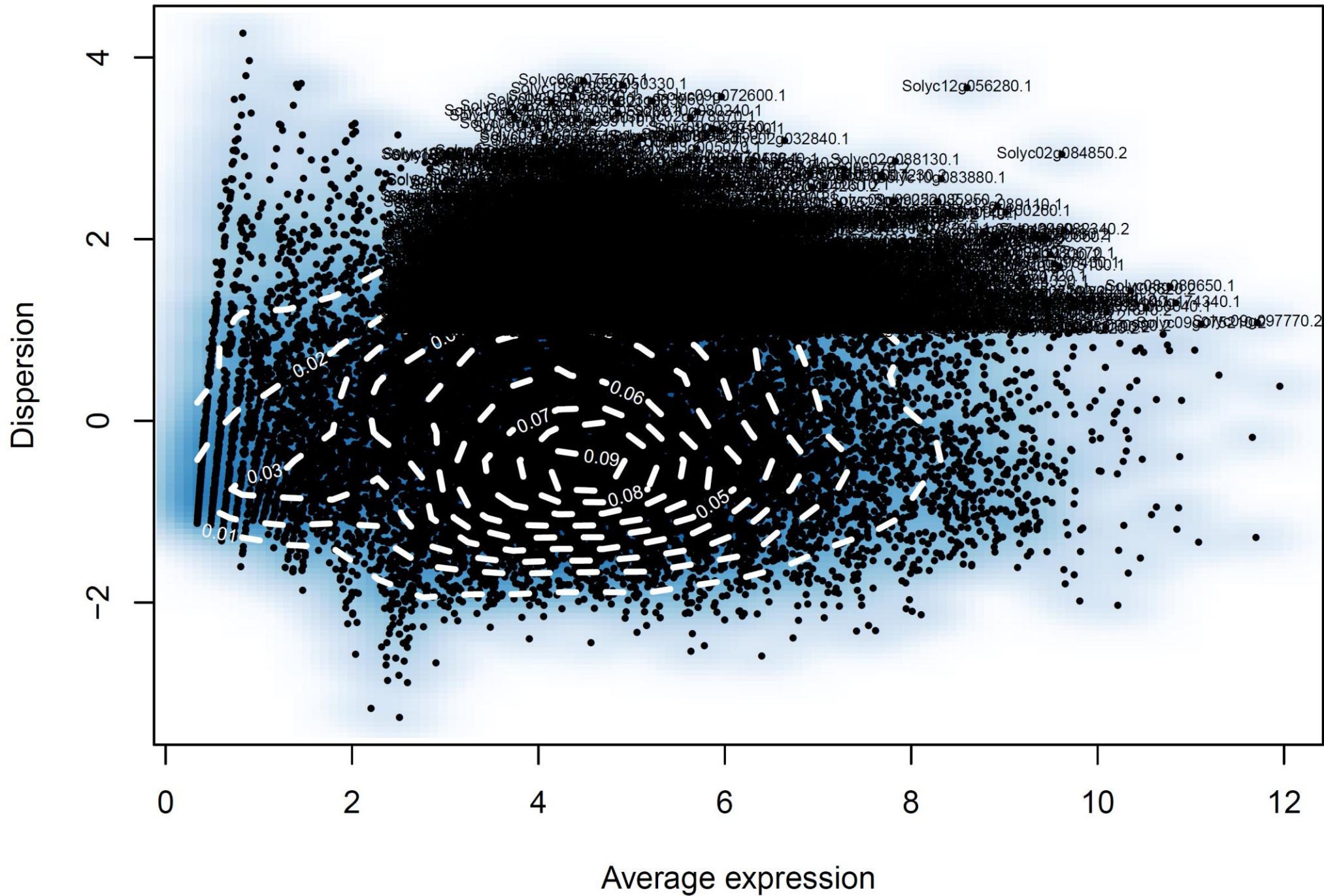

Supplement: Supplementary file 3 — Fig. S2. Dispersion pattern of gene expression data among the different RNA-seq samples. Dots labeled with names indicate the variable genes selected. (PDF 356 kb) [file 12870_2019_1976_MOESM3_ESM.pdf]

Scale Free Topology Model Fit, signed  $R^2$

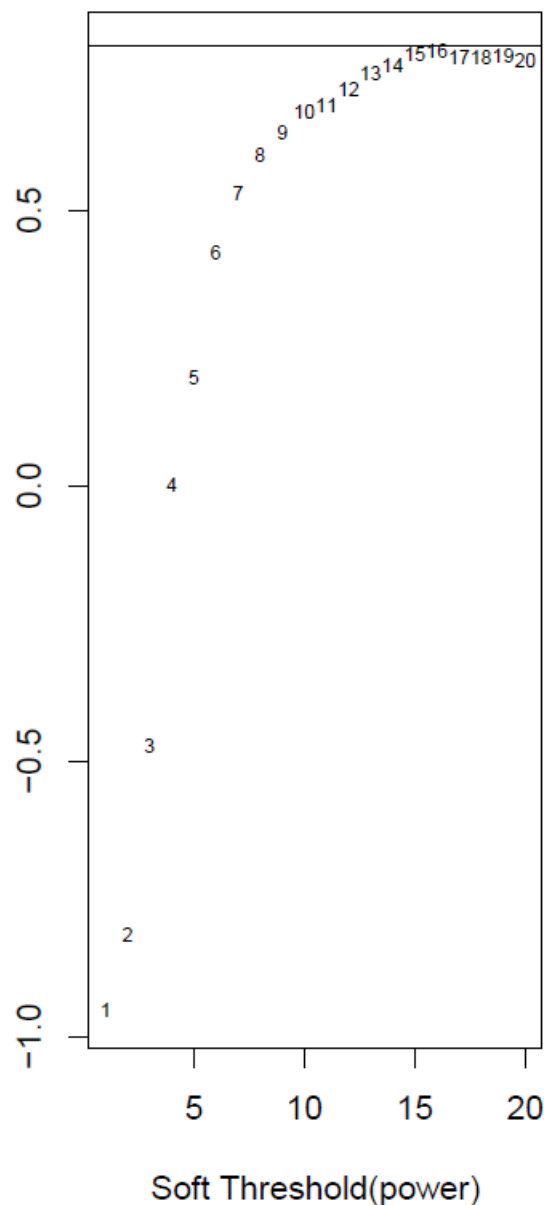

Mean Connectivity

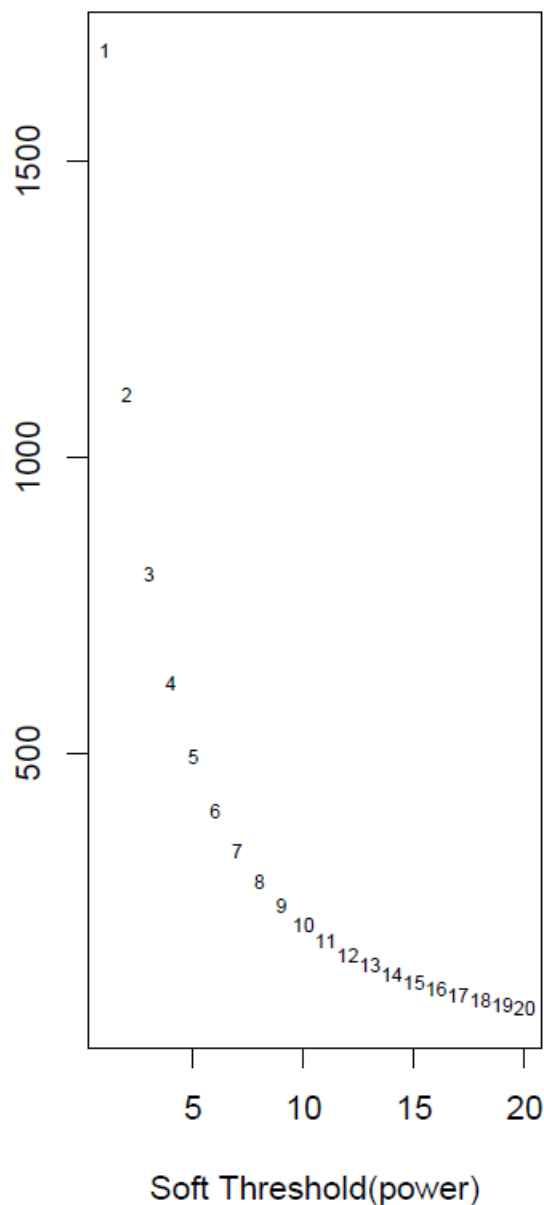

Supplement: Supplementary file 4 — Fig. S3. Soft thresholds chosen by applying the approximate Scale-free Topology Criterion. (PDF 38 kb) [file 12870_2019_1976_MOESM4_ESM.pdf]
